# Drug-Induced Oxidative Stress and Toxicity

**DOI:** 10.1155/2012/645460

**Published:** 2012-08-05

**Authors:** Damian G. Deavall, Elizabeth A. Martin, Judith M. Horner, Ruth Roberts

**Affiliations:** Safety Assessment, AstraZeneca, Alderley Park, Macclesfield SK10 4TG, UK

## Abstract

Reactive oxygen species (ROS) are a byproduct of normal metabolism and have roles in cell signaling and homeostasis. Species include oxygen radicals and reactive nonradicals. Mechanisms exist that regulate cellular levels of ROS, as their reactive nature may otherwise cause damage to key cellular components including DNA, protein, and lipid. When the cellular antioxidant capacity is exceeded, oxidative stress can result. Pleiotropic deleterious effects of oxidative stress are observed in numerous disease states and are also implicated in a variety of drug-induced toxicities. In this paper, we examine the nature of ROS-induced damage on key cellular targets of oxidative stress. We also review evidence implicating ROS in clinically relevant, drug-related side effects including doxorubicin-induced cardiac damage, azidothymidine-induced myopathy, and cisplatin-induced ototoxicity.

## 1. Introduction

**Figure 1:**
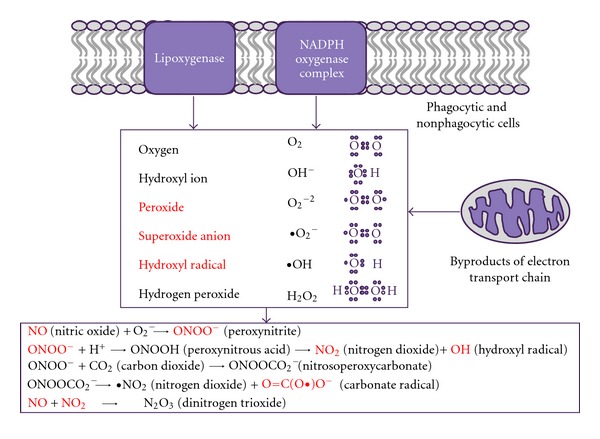
Reactive oxygen species: main forms and sources. Reactive oxygen species occur mainly as byproducts of the mitochondrial respiratory chain but can also originate from the activities of NADPH and lipoxygenase. Once released, reactive oxygen species can react with NO leading to the generation of reactive nitrogen species. Molecules with unpaired electron free radicals are shown in red.

Chemically reactive molecules containing oxygen are termed reactive oxygen species (ROS). Reactivity may be due to the presence of unpaired electrons, but there are also reactive nonradical species such as hydrogen peroxide (H_2_O_2_). Examples of ROS are shown in Figure 1 and include peroxides and free oxygen ions generated during the normal metabolism of oxygen via diverse enzymatic pathways. ROS can be generated from a variety of sources both endogenous and exogenous. One of the main sources of ROS within the cell is the mitochondrion, where the superoxide radical ^•^O_2_^−^ is produced as a byproduct of normal oxidative phosphorylation. Although not the focus of this paper, in addition to driving the generation of ROS, ^•^O_2_^−^ is highly reactive with nitric oxide (NO), generating reactive nitrogen species (RNS) such as peroxynitrite and further downstream nitrogen species, including NO, peroxynitrite, and nitrogen dioxide (see Figure 1), via the activity of enzymes such as inducible nitric oxide synthase 2 (NOS2) and NADPH oxidase (NOX).

ROS have roles in normal cell signaling and homeostasis [1]. For example, in the vasculature, ^•^O_2_^−^ may act to limit the duration of the response to NO, a key mediator in vascular functions, including regulation of smooth muscle tone and blood pressure, platelet activation, and vascular cell signaling [2]. However, beyond normal physiological roles, excessive production of ROS can occur in response to such stressors as toxicant exposure, radiation damage, and disease, resulting in local oxidative stress and consequent adaptive responses.

Cells have a variety of defense mechanisms that intercept free radicals to prevent or limit intracellular damage and ameliorate the harmful effects of ROS, including low-molecular-weight antioxidants (such as ascorbic acid, vitamin E, and glutathione) and antioxidant enzymes (such as thioredoxins, superoxide dismutase (SOD), catalase, and glutathione peroxidase). A key example of the latter is mitochondrial manganese superoxide dismutase (MnSOD), which converts superoxide radicals to hydrogen peroxide, which is further broken down into water by peroxidases [3]. As a consequence of these activities, physiological levels of ROS are low. However, with heightened levels of ROS, defense systems can be overwhelmed resulting in cellular damage. Normally functioning cells can sustain and tolerate background levels of damage, but if an imbalance occurs, then cellular damage will increase. This damage may result from significant modification of intracellular targets such as DNA, proteins, and lipids and may modulate survival signaling cascades. At the molecular level, the extent of damage depends on many factors including the site of ROS production, reactivity of the target, and the availability of metal ions. Modified proteins and lipids can be removed by normal cellular turnover, but DNA damage requires specific repair mechanisms. When mitochondrial DNA is the target of oxidation, it can lead to mutations, rearrangements, and transcriptional errors that impair important mitochondrial components, leading to more oxidative stress and eventual cell death. Molecular modifications in surviving cells can cause alterations in gene expression, and, depending on the severity and duration of ROS exposure, prosurvival or proapoptotic response pathways may be activated.

Oxidative-stress-induced damage to DNA and macromolecules is associated with the onset and development of many diseases including cardiovascular disease, neurological degenerations (e.g., Alzheimer's disease, ischemic stroke), and cancer, as well as the normal ageing processes. Tumour cells have high levels of ROS, and studies have shown elevated levels of oxidative stress and/or oxidative DNA damage in human malignancies relative to normal cells [4, 5]. Generation of ROS at complex I of the electron transport chain (ETC), known as “complex I syndrome,” has been linked to age-associated modifications in the central nervous system [3, 6]. Conversely, the production of ROS and RNS is a key feature of some desirable immunological responses where, in response to activation by pathogens, phagocytes produce reactive species, including superoxide, nitric oxide, and peroxynitrite that can damage infected cells.

**Figure 2:**
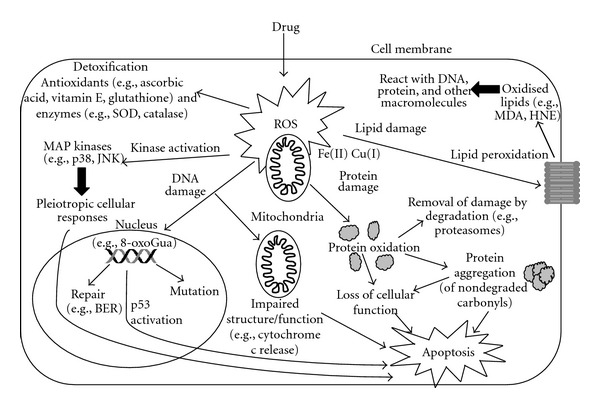
The main effects of drug-induced oxidative stress in cells. Increases in intracellular ROS may result in DNA damage, oxidation of lipids and proteins. MAP kinase signaling pathways are key mediators of the cellular response.

In addition to association with disease states, there is clear evidence to implicate drug-induced oxidative stress as a mechanism of toxicity in numerous tissues. As illustrated in Figure 2, ROS have effects on key cellular targets, namely, DNA, lipid, and protein macromolecules (see Figure 2). ROS may damage these critical cellular components at the molecular level, with consequent effects of ROS on cell survival mediated by kinase cascades. These factors may have a key role in initiating cell death in response to oxidative insult.

## 2. Cellular Targets of ROS

### 2.1. DNA Damage

In a cell, an estimated 10^5^ oxidative lesions are formed everyday [7]. Oxidation of DNA leads to the formation of lesions including oxidized bases (purines and pyrimidines), abasic sites (also called apurinic/apyrimidinic (AP) sites), and DNA single- and/or double-strand breaks. Guanine is the most susceptible DNA base because of its low oxidation potential, and there are multiple oxidized guanine products [8]. Two of the most common modifications are 8-oxo-7,8-dihydroguanine (8-oxoGua) and 2,6-diamino-4-hydroxy-5-formamidopyrimidine (FapyGua), which originate from the addition of the hydroxyl radical to the C8 position of the guanine ring, producing an 8-hydroxy-7,8-dihydroguanyl radical, which can then be either oxidized to 8-oxoGua or reduced to give the ring-opened FapyGua [9]. The corresponding adenine modifications, 8-oxo-7,8-dihydroadenine (8-oxoAde) and 4,6-diamino-5-formamidopyrimidine (FapyAde), are also generated. Further purine lesions produced by oxidative stress include 2-hydroxyadenine (2-OH-Ade), xanthine, and hypoxanthine, which are products of the deamination of guanine and adenine, respectively, and 8,5′-cyclo-2′-deoxyguanosine (cyclo-dG) and the adenine equivalent cyclo-dA [10].

Of all the DNA oxidation products, 8-oxoGua is the most abundant, stable, and well studied and therefore often used as a biomarker of oxidative stress. It is a strongly promutagenic lesion, since it promotes the mismatched incorporation of dATP instead of dCTP opposite the lesion during replication, inducing a GC to TA transversion [11]. It is estimated that there is a steady-state level of approximately one 8-oxoGua lesion per 10^6^ normal nucleosides [12]. ROS can also react with dGTP in the nucleotide pool to form 8-oxoGua. Hence, during DNA replication, 8-oxoGua can be incorporated into DNA opposite dC or dA on the template strand, resulting in AT to CG transversions [13]. 8-oxoAde is far less studied than 8-oxoGua but is reported to be 3- to 4-fold less mutagenic than 8-oxoGua in a mammalian system [14].

Hydroxyl radicals react with pyrimidines (thymine and cytosine) at positions 5 or 6 of the ring producing several lesions, the most abundant of which are 5,6-dihydroxy-5,6-dihydrothymine (thymine glycol) and 5,6-dihydroxy-5,6-dihydrocytosine (cytosine glycol). Products of cytosine may deaminate and dehydrate giving uracil glycol, 5-hydroxycytosine (5-OH-Cyt), and 5-hydroxyuracil (5-OH-Ura). Of the pyrimidine-derived lesions, 5-OH-Cyt and 5-OH-Ura are potentially premutagenic lesions leading to GC to AT transitions and GC to CG transversions [15]. Techniques and methods for measuring oxidative DNA damage (typically 8-oxoGua) include high-performance liquid chromatography (HPLC), gas chromatography mass spectrometry (GC-MS), liquid chromatography-tandem mass spectrometry (LC-MS/MS), and single-cell gel electrophoresis (comet) assay [16].

Several DNA repair pathways can protect from the deleterious effects of oxidative DNA damage. The primary repair pathway for oxidative base lesions, including 8-oxoGua, is base excision repair (BER) [17]. BER recognizes and repairs oxidized bases, AP sites, DNA single-strand breaks, alkylated bases, deaminated bases, and base mismatches [4]. BER uses specific glycosylases to release the damaged base leaving an AP site, followed by the processes of abasic site priming, gap filling, and DNA ligation, which are common irrespective of the glycosylase used. Several DNA glycosylases specifically recognize and remove 8-oxoGua in human cells, the primary being 8-oxoGua glycosylase (OGG1) [14]. Human OGG1 also recognizes and excises several other oxidative lesions, including FapyGua and 8-oxoAde [11]. Formamidopyrimidine-DNA glycosylase (Fpg, MutM) is specific for oxidized purines, including 8-oxoGua, FapyGua, and FapyAde and other ring-opened purines [18, 19]. Endonuclease III recognizes oxidized pyrimidines, including thymine glycol and uracil glycol [20, 21]. Other glycosylases which recognize oxidative damage include NTH, NEIL, and MYH [10]. Cyclo-dG and cyclo-dA are likely substrates for nucleotide excision repair (NER), rather than BER. NER removes an oligonucleotide containing the lesion, not just the damaged base. The process is complex and involves multiple lesion recognition and incision proteins, DNA synthesis and ligation [10]. FapyGua and FapyAde are potential precursors of apurinic sites, since the opening of the imidazole ring is known to increase the hydrolytic lability of their N-glycosidic bonds. This is very common and can occur spontaneously or enzymatically via DNA glycosylases during BER [22]. AP sites are not considered lethal unless in high levels and, if present, are expected to block DNA polymerases [23].

High levels of DNA damage may exceed the cellular repair capacity, generating mutations and triggering apoptosis. It has been shown that the tumour suppressor p53 is an important regulator of the cellular response to ROS-induced DNA damage. p53 is activated as a transcription factor and induces target genes involved in cell cycle arrest, DNA repair, and apoptosis. For example, p53 participates in sensing oxidative DNA damage and modulating BER function in response to persistent ROS stress [24]. Under severe ROS stress, high levels of DNA damage cause persistent accumulation/activation of p53 which leads to induction of apoptosis in the damaged cells. Hence, cells with high levels of DNA damage are eliminated maintaining the genetic integrity of the whole cell population.

### 2.2. Lipid Damage

Oxidative stress can induce radical-mediated damage to cellular biomembranes resulting in lipid peroxidation, which converts unsaturated lipids into polar lipid hydroperoxides. Lipid peroxidation can also lead to the generation of a variety of oxidized products including reactive electrophiles, such as epoxides and aldehydes, which are capable of modifying DNA, protein, and other macromolecules. Examples include malondialdehyde (MDA), 4-hydroxy-2-nonenal (HNE), 2-propenal (acrolein), and isoprostanes, which can be measured as an indirect index of oxidative stress [25]. MDA reacts with nucleic acid bases to form dG, dA, and dC adducts [26] and is mutagenic [27]. Lipid peroxidation may impair normal cell function by increasing membrane fluidity, inactivating membrane-bound receptors or enzymes, and promoting efflux of cytosolic solutes [28]. It has also been implicated in human diseases [25] such as cancer [29], diabetes [30], acute lung injury [31], Alzheimer's disease [32], and Parkinson's disease [33]. Stable protein adducts formed by MDA are immunogenic, and the serum concentration of autoantibodies against MDA-modified lysine residues is reportedly associated with the burden of, and may predict the progression, of atherosclerosis and myocardial infarction [34].

### 2.3. Protein Damage

Oxidation-sensitive proteins include phosphatases, kinases, transcription factors, and metabolic enzymes, so consequently, protein oxidation can have a major impact on cellular homeostasis by directly affecting cell signaling, cell structure, and enzymatic processes such as metabolism. Certain proteins are more susceptible to oxidation than others. Susceptibility factors include the relative content of oxidation-sensitive amino acid residues, for example, protein-tyrosine phosphatases, mitogen-activated protein kinase kinase 6, and the nuclear factor I transcription factors, all contain oxidation sensitive cysteines [35–37]. Other susceptibility factors include the presence of metal-binding sites, protein localisation in the cell, molecular conformation, and rate of degradation [28]. Newly synthesized proteins may be most prone to oxidative damage, indicating that complete folding and incorporation into protein complexes offers protection from oxidation-driven degradation [38]. Oxidation-sensitive proteins are often associated with particular metabolic pathways or functions such as energy metabolism (e.g., glyceraldehyde 3-phosphate dehydrogenase (GAPDH) and creatine kinase), mitochondrial proteins, chaperones, and members of the ubiquitin-proteasome system.

Oxidation of metabolic enzymes such as those involved in glycolysis and the citric acid cycle may contribute further to oxidative stress. Inhibition of GAPDH accelerates glycolytic processes resulting in a loss of ATP production overall [39]. It has been reported that increased H_2_O_2_ exposure results in inhibition of GAPDH [40]. Inhibition of GAPDH via oxidative stress may also be involved in modulation of apoptosis and cell signaling [41]. Enzymes in the citric acid cycle may also be important in cellular dysfunction caused by oxidative stress. Under various oxidative conditions, the activities of several of these enzymes have been shown to be reduced [42].

Protein oxidation can be induced in a number of ways including metal-catalysed oxidation, oxidation-induced cleavage, amino acid oxidation, and the conjugation of lipid peroxidation products [43]. Of these, the most common mechanism for inducing protein oxidation is metal-catalysed oxidation. This requires metal ions such as Fe(II) or Cu(I) to bind to metal binding sites within proteins, which then react with H_2_O_2_ to generate hydroxyl radicals that attack neighbouring amino acid residues [44]. A second mechanism is ROS-induced cleavage of peptide bonds following the generation of alkoxyl radicals by either the diamide or *α*-amidation pathways. These pathways lead to different peptide fragments at the site of cleavage, with a diamide and isocyanate formed via the diamide pathway, and an amide and *N*-*α*-ketoacyl formed via the *α*-amidation pathway [45]. Amino acids can be modified directly via side chain reaction with ROS. The most sensitive amino acids are those with aromatic side chain groups, for example, phenylalanine and histidine, and those containing sulfhydryl groups, for example, methionine and cysteine. Cysteine is also vulnerable to oxidant-induced cross-linking. Oxidative modifications of sulfur-containing amino acids can be reversible. For example, the oxidation of methionine, which is one of the most oxidation-prone amino acid residues, can be reversed by methionine sulfoxide reductase enzymes.

Removing oxidized and damaged proteins from cells is essential for homeostasis. Oxidized proteins are degraded via the proteasome and lysosomal pathways. The proteasome is the major pathway for regulating mildly oxidized proteins by degrading them to short peptides. Ubiquitination allows the target protein to be recognised by the proteasome and targeted for protein degradation. Ubiquitin is then recycled. Oxidised proteins can also be degraded by the proteasome in the absence of ubiquitin [46].

During oxidation, several amino acid residues (e.g., arginine, proline, histidine and lysine) irreversibly form carbonyl products, the most commonly measured product of protein oxidation in biological samples. Highly sensitive methods are available for carbonyl detection including HPLC with electrochemical detection or mass spectrometry, enzyme-linked immunosorbent assay (ELISA), and immunohistochemistry [47]. Carbonyl formation provides a marker for damaged proteins to be inactivated by proteasomal degradation. If not degraded, carbonyls can further react with the *α*-amino groups of lysine residues, leading to the formation of intra- or intermolecular cross-links which can promote the formation of high-molecular-mass aggregates [43]. Aggregates are extremely resistant to proteolysis and can act as inhibitory compounds towards both the proteasome and lysosome degradation pathways. Protein aggregates can be highly cytotoxic, altering cell functions and leading to necrosis or apoptosis. Aggregates are a feature of age-related disorders, such as Parkinson's disease and Alzheimer's disease, and cancer [28].

### 2.4. Modulation of Kinase Signaling

Protein kinases mediate the activation of cellular signaling cascades controlling growth, proliferation, and survival in response to extracellular and intracellular stimuli. It is well established that multiple kinase signaling pathways are affected by ROS ([48] for review) and kinase activation is critical in detecting oxidative stress and transducing signals to initiate a cellular response.

Key mediators of the biological effects of ROS include mitogen-activated protein kinases (MAPKs), apoptosis signal-regulating kinase 1 (ASK1), p38 and c-Jun N-terminal kinases (JNK), as well as the phosphotidylinositol 3-kinase (PI3K)/protein kinase B (Akt) pathway, tyrosine kinases (e.g., Jak, Src, EGFR, PGDFR), and protein kinase C. Interactions between kinases generate a wide range of possible signaling cascades, the output of which may ultimately impact on homeostasis via both cell survival and cell death signals.

Kinase-dependent proapoptotic signaling is one important response to oxidative stress, and there are many pathways by which this response can occur. For example, ASK-1 may act as an important mediator of ROS-induced apoptosis. This MAP kinase kinase kinase is activated by cellular stress, particularly oxidative stress, and can phosphorylate and activate both JNK and p38 proteins to induce apoptosis via mitochondria-dependent mechanisms involving cytochrome c release and caspase-3/-9 activation [49, 50]. Under normal conditions, the N-terminal region of ASK1 is bound by thioredoxin (Trx) and kinase activity is inhibited. However, under conditions of oxidative stress, oxidized Trx dimerises and dissociates from ASK1, which oligomerises and is capable of autophosphorylation and activation of downstream MAPK proteins, JNK, and p38. In this cascade, Trx acts as a redox sensor to promote MAPK-mediated apoptosis in response to ROS, which contrasts with its intrinsic antioxidant role of facilitating protein reduction by cysteine thiol-disulphide exchange. Activation of JNK and p38 is associated with proapoptotic responses. Activated JNK may translocate to the nucleus, where it phosphorylates and activates c-Jun. c-Jun, as part of the AP-1 transcriptional complex, may regulate expression of proapoptotic genes such as members of the TNF-*α* family. Also, JNK may translocate to mitochondria to activate proapoptic proteins, such as members of the “BH3-only” subgroup of the Bcl-2 family (e.g., Bid, Bim), or suppress/antagonize the activity of antiapoptotic Bcl-2 and Bcl-x_L_ proteins (reviewed in [51]). This may activate caspase signaling cascades by promoting release of cytochrome c from the inner mitochondrial membrane. JNK (and other MAPKs) also phosphorylates the tumour suppressor protein p53, a key mediator of proapoptotic responses when accumulated in stressed cells. Levels of p53 are usually kept low through Mdm2-dependant degradation (Mdm2 protein functions as a ubiquitin ligase and an inhibitor of p53 transcriptional activation), but phosphorylation both disrupts Mdm-2 binding, leading to accumulation, and allows p53 to facilitate transcriptional upregulation of proapoptotic genes.

Although the consequences of oxidative stress are considered primarily deleterious, activation of kinase signaling cascades by ROS may be protective and promote cell survival. An interesting example is activation of the PI3K/Akt pathway. It is well established that PI3K activation and subsequent phosphorylation/activation of Akt promote cell survival, for example, in response to stimulation by peptide growth factors (reviewed in [52, 53]). Mechanistically, Akt promotes cell survival via negative regulation of proapoptotic Bcl2 family proteins, including BAD which is phosphorylated by the PI3K/Akt pathway *in vitro* and *in vivo* [54]. In addition to prosurvival signaling by growth factors via PI3K/Akt, it has been shown that ROS may contribute to cell survival, and may prevent damage caused by oxidative stress through activation of PI3K and Akt. For example, phosphorylation and activation of Akt occur in response to NAPDH oxidase-dependent ROS accumulation in monocytes, and this is associated with induction of cellular survival [55]. Furthermore, PI3K activation may initiate expression of antioxidant genes by mediating nuclear translocation of the transcription factor Nrf2 which may bind, in complex with Maf, to antioxidant response elements (AREs) and initiate expression of antioxidant genes [56]. It has recently been shown that, in response to antioxidant flavonoid, expression of MnSOD is increased via activation of the PI3K/Akt signaling pathway and upregulation of ARE-mediated, Nrf2-dependent gene expression [57].

## 3. Drug-Induced Oxidative Stress as a Mechanism of Toxicity

Drug-induced oxidative stress is implicated as a mechanism of toxicity in numerous tissues and organ systems, including liver, kidney, ear, and cardiovascular and nervous systems. Well-characterized drugs associated with adverse events to which oxidative stress may contribute, including examples of cancer therapies, non-steroidal anti-inflammatory drugs (NSAID), antiretroviral agents, antipsychotics, and analgesics, as illustrated in Table 1. Though by no means, a comprehensive list, the examples in Table 1 serve to illustrate the potential for mechanistically distinct therapies to cause diverse toxicities with oxidative stress as a key contributor.

**Figure 3:**
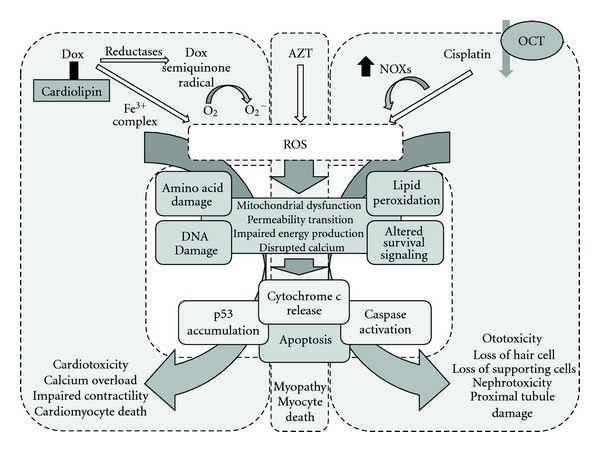
Molecular and cellular events by which oxidative stress in response to Dox, AZT, and cisplatin may result in toxicity. Dox, AZT, and cisplatin accumulation in cells may result in elevations in intracellular ROS. Dox may accumulate in cardiac cells by association with cardiolipin and generate ROS via reduction of molecular oxygen by the semiquinone free radical or by an iron II-Dox radical. Cisplatin may be transported into cells via the OCT transporters (e.g., in renal tubule cells) and elevate ROS levels via induction of NOXs. At the molecular level, ROS damage amino acids, lipid, and DNA. Mitochondrial dysfunction and associated alterations in energetics, together with effects on survival/apoptotic signaling cascades may lead to a proapoptotic response. These common mechanisms may be key to Dox-dependent cardiotoxicity, AZT-dependent skeletal myopathy, and cisplatin-dependent nephrotoxicity and ototoxicity described further in Section 3.

The extent to which mechanisms of drug-induced oxidative stress have been characterized varies. Metabolism of a drug may generate a reactive intermediate that can reduce molecular oxygen directly to generate ROS, as discussed below for doxorubicin. Chlorpromazine is an interesting example as photoactivation in skin is considered likely to lead to cutaneous phototoxicity (sunburn-like reaction and hyperpigmentation), which is a well-know adverse event associated with this compound [58]. Photodechlorination converts chlorpromazine to an excited state with subsequent energy transfer to molecular oxygen and generation of both excited singlet oxygen and superoxide species. These species may then react with DNA and macromolecules as described above and trigger adaptive or toxic responses in the skin as a result. For other drugs, there is evidence of elevation in cellular ROS in response to drug exposure, and evidence implicates ROS and oxidative stress in toxicity even if the mechanisms by which ROS are generated are characterized less fully. In this section, we discuss further the evidence for involvement of oxidative stress in drug-induced toxicities, using the examples of doxorubicin, azidothymidine, and cisplatin. In Figure 3, the common mechanisms by which oxidative stress in response to treatment with these drugs can lead to tissue-specific toxicities are presented.

### 3.1. Doxorubicin

Doxorubicin (Dox) is an anthracycline antibiotic used in numerous chemotherapy regimens to treat haematological and solid tumours. The antineoplastic activity is mediated by intercalation of DNA, preventing replication and protein synthesis, and inhibition of topoisomerase II, preventing topoisomerase II-dependent religation after double-strand breakage [59, 60]. Though effective as an anticancer drug, dose-dependent cardiotoxicity (characterised as either acute or early-/late-onset chronic progressive cardiomyopathy) is a well-described side effect of Dox therapy and is a major limitation to its use.

Acute cardiac events with Dox are now rare and usually reversible but may include acute tachycardia, hypotension, and heart failure as a consequence of high doses. Early onset chronic effects typically occur during treatment or within 1 year, whilst late onset chronic effects, for example cardiac dilation, may occur long after treatment with Dox has stopped and may not present clinically until 20 years after starting Dox therapy [61]. Chronic effects may lead to fatal congestive heart failure.

The mechanisms underlying effects on cardiac tissue have been investigated intensively. Free radical formation, lipid peroxidation, mitochondrial dysfunction, altered calcium handling, DNA damage, p53 accumulation and activation of proapoptotic signaling cascades/inhibition of survival signaling have all been implicated. Although these mechanisms are not fully elucidated and are multifactorial, there is substantial evidence to support a key role for Dox-induced oxidative stress in clinically relevant cardiotoxicity.

#### 3.1.1. Formation of ROS by Dox

Dox may generate ROS by more than one mechanism [62, 63]. Reduction of Dox by one electron via mitochondrial reductases may generate anthracycline semiquinone free radicals [64]. Under aerobic conditions, these are unstable and readily reduce molecular oxygen to the ROS superoxide anion and H_2_O_2_ [65]. Reactions between iron and Dox may also generate ROS. Redox reactions subsequent to interaction of Dox with iron (III) may generate an iron II-Dox free radical, capable of reducing molecular oxygen.

The oxidative stress resulting from increased free radical generation in cardiomyocytes may lead to multiple adverse effects including energetic imbalance, perturbations in mitochondrial function, activation of stress-related signaling pathways (such as p38 and JNK), p53 accumulation, and, ultimately, cell death. To illustrate, studies in mice have shown that Dox induces elevations in ROS, DNA damage, activation of ataxia telangiectasia-mutated (ATM) kinase signaling, accumulation of p53, and cardiomyocyte death. Similar Dox-induced events, when measured in cultured mouse cardiomyocytes, are not seen in the presence of a free radical scavenger. It is interesting to note that inhibition of ATM kinase (which signals DNA damage) reduced Dox-induced accumulation of p53, suggesting a link between DNA damage and apoptosis in response to Dox. Furthermore, in transgenic mice deficient in p53 or overexpressing Bcl-2 in cardiac tissue, Dox cardiac damage, including contractile dysfunction and myocyte apoptosis, was attenuated [66].

#### 3.1.2. Role of Mitochondria in Dox-Induced Cardiotoxicity

A possible contributor to the sensitivity of cardiomyocytes to Dox-induced damage may be the high affinity of Dox for the mitochondrial phospholipid cardiolipin [67], localised to the inner mitochondrial membrane and critical to mitochondrial structure, function and energy metabolism in cardiomyocytes. As cardiac tissue is dependent on oxidative metabolism, it is rich in mitochondria. Thus, disproportionate accumulation of Dox in mitochondria via interaction with cardiolipin could lead to a significant enhancement of ROS generation in cardiac tissue. Mitochondrial swelling, depolarisation, perturbations of energetics, and dysregulation of mitochondrial calcium signaling have all been reported following exposure to Dox *in vitro* or *in vivo* [68–70]. The consequent disruption of calcium signaling pathways and calcium-dependent ATP synthesis that could result from perturbations in mitochondrial structure and function may be key contributors to toxicity in cardiomyocytes, via induction of apoptosis [71]. In addition, to further illustrate the potential for mitochondrion-dependent apoptosis in response to Dox, single doses of Dox to rats are associated with release of cytochrome c (which binds cardiolipin in the mitochondrial membrane) and increases in proapoptotic caspase 3 activity [72] which could then initiate apoptotic degradation.

Clinical data suggest potential for exacerbation of ROS-mediated cardiac toxicity with therapies that perturb the natural cellular response to oxidative stress, even if such therapies may not cause significant ROS-mediated damage themselves. Trastuzumab is a humanized monoclonal antibody against human epidermal growth factor receptor 2 (HER2). Trastuzumab therapy is associated with cardiovascular toxicity in HER2-positive breast cancer when administered in combination with anthracyclines. As a response to oxidative stress with Dox, the myocardial survival signaling pathway is activated. A key component of this is stimulation of HER2 which would serve to protect cardiomyocytes by blocking Bcl-x_L_/caspase 3-mediated apoptosis. However, this natural adaptive response is inhibited in the presence of trastuzumab, and the proapoptotic response to ROS-mediated Dox toxicity in cardiomyocytes would be exacerbated. Analysis of data from phase 3 clinical trials with trastuzumab illustrated the incidence of symptomatic cardiac disease was more frequent (and more severe) in patients with a previous history of Dox therapy or in patients given trastuzumab and Dox in combination [73–75].

#### 3.1.3. Prevention of Dox-Induced Cardiovascular Damage

As oxidative stress is a consequence of elevated ROS, the observation that attenuation of Dox cardiotoxicity can be achieved by elevating antioxidants is further support for the role of oxidative stress as a mediator of Dox toxicity in the heart.

Extensive data have been generated in numerous model systems showing that administration of antioxidants protects cardiomyocytes from Dox-induced damage. The range of molecules explored is diverse, including plant extracts, vitamins C and E, the beta-blocker carvedilol, L-carnitine, n-acetylcysteine, coenzyme Q10, and dexrazoxane [76, 77]. Results from *in vitro* and nonclinical *in vivo* studies are often compelling and show a decrease in ROS-induced cardiomyocyte damage. For example, when administered to rats orally, carvedilol prevented Dox-induced lipid peroxidation and cardiomyopathy [76]. Recently, it has been reported that the sedative 2,6-diisopropylphenol (propofol) attenuates both oxidative stress and cellular apoptosis in Dox-treated cultured rat neonatal cardiomyocytes [78]. In this study, propofol countered Dox-induced ROS production, disruption of mitochondrial membrane potential, cytochrome c release, caspase 3 activity, and apoptosis. Finally, it is interesting to note that *in vitro* and *in vivo* (in mice) Dox-induced damage to cardiac cells (including DNA damage, apoptosis, and contractile dysfunction) is ameliorated by administration of a statin (pitavastatin, a 3-hyroxy-3-methylglutaryl-CoA reductase inhibitor), through its antioxidant effect and inhibition of the guanosine triphosphatase Rac1, a regulator of NAPDH oxidase activity [66].

Whilst nonclinical data illustrate a link between antifree radical treatment and attenuation of Dox-induced cardiomyopathy, the picture is less clear clinically, with administration of many antioxidant molecules failing to show compelling cardioprotective effects in Dox-treated patients [79]. However, evidence supports the use of Dexrazoxane, a bis-dioxopiperazine compound approved by FDA and EMA to reduce the incidence or severity of cardiomyopathy in breast cancer patients who have received Dox at 300 mg/m^2^. Dexrazoxane chelates intracellular iron, which would inhibit the iron-dependent production of free radicals described above [80]. Analysis of randomised clinical studies of dexrazoxane with doxorubicin has indicated a decrease in occurrence of cardiotoxicity compared with doxorubicin alone. Mainly, data suggest the efficacy of Dox is unaffected [81]. These data support the assertion that oxidative stress in cardiomyocytes in response to Dox exposure is implicated in clinical cardiotoxicity observed with this antineoplastic agent anthracycline.

### 3.2. Azidothymidine

As described above, in addition to generating ROS following drug exposure, mitochondria are also a toxicity target of oxidative stress. As a further example, evidence suggests that mitochondrial dysfunction due to oxidative stress is implicated in toxicities observed following long-term administration of azidothymidine (AZT). AZT was the first antiretroviral drug approved for treatment of HIV. As a potent nucleoside reverse transcriptase inhibitor, AZT prevents DNA synthesis from viral RNA and thus prevents viral replication. AZT is administered chronically in combination with other antiretroviral drugs in “Highly Active Antiretroviral Therapy” regimens [82]. Unfortunately, chronic administration of AZT is associated with several side effects including neuropathy, cardiac dysfunction, and skeletal myopathy. Clinically, in addition to the myopathy associated with HIV infection, AZT causes pathological changes in skeletal muscle, consistent morphologically with mitochondrial abnormality [83]. In cultured human muscle cells *in vitro*, AZT decreased proliferation, increased lactate production, and decreased cytochrome c oxidase activity [84], indicating further the potential for AZT to affect mitochondrial function.

Transgenic mice under- or overexpressing SOD have been used to characterise AZT-induced oxidative stress *in vivo*. Depletion of SOD was associated with enhanced cardiomyopathy, whilst the heart was protected in mice overexpressing SOD or expressing mitochondrion-targeted catalase. This implicates hydrogen peroxide, as an oxidative product of dismutation, in AZT-induced toxicity. More recently, direct detection and quantification of ROS and RNS in response to AZT have been reported using a mouse macrophage model system, which enabled identification of specific reactive species. In this study, cells responded to incubation with AZT by releasing reactive species including peroxide and peroxinitrate [85]. Interestingly, thymidine alone did not increase the release of ROS/RNS in the same way, suggesting the azido moiety is important in oxidative stress. This finding is supported by studies in human aortic endothelial cells, in which oxidative stress, decreased mitochondrial membrane potential, increases in lactate release (an indicator of impaired mitochondria producing energy by cytosolic glycolysis), and cell death were observed when incubated for several weeks with AZT, but not when incubated with d4T (stavudine) which lacks the azido group [86].

### 3.3. Cisplatin

Toxicities related to drug-induced oxidative stress occur in multiple tissues, and it is interesting to note *cis*-diamminedichloroplatinum (cisplatin) as an example of a drug that exhibits multiorgan toxicity with redox imbalance as a possible mechanism. Cisplatin is an antineoplastic agent used in the treatment of testicular, bladder, lung, gastrointestinal, and ovarian cancers. Clinically, ototoxicity, neurotoxicity (peripheral neuropathy), neurotoxicity, and renal toxicity (nephrotoxicity) have been described, and it has been suggested that for some toxicities there is an association between residual platinum levels and severity of toxicity 5 to 20 years after therapy [87]. There is evidence to support a role for cisplatin-induced oxidative stress in each of these adverse effects.

Nephrotoxicity limits clinical use of cisplatin and primarily affects the S3 segment of the proximal tubule (PT) [88]. It has been shown that cisplatin enters cells via the organic cation transporter (OCT) 2 [89], which in the human kidney is expressed predominantly at the basolateral surface of PT cells. Transport via OCT2 may be responsible for accumulation of cisplatin within the PT. Indeed, it has been shown that OCT1 and OCT2 knockout mice are protected against severe cisplatin-induced renal tubular damage. Furthermore, a single-nucleotide polymorphism in the OCT2 gene *SLC22A2* was associated with reduced cisplatin-induced nephrotoxicity in patients [90].

Both *in vitro* and *in vivo*, cisplatin has been shown to increase oxidative stress by increasing levels of superoxide anion, H_2_O_2_, and hydroxyl radical [91, 92]. Again, the potential for antioxidants and ROS scavengers to protect against cisplatin-induced nephrotoxicity in experimental models supports the involvement of oxidative stress in this toxicity [93]. The translation of oxidative stress to renal impairment has been demonstrated in rodents *in vivo*. Perturbations in mitochondrial function and integrity (as suggested by lipid peroxidation), depletion of key antioxidants, changes in membrane potential, changes in calcium handling, caspase 3 activation and apoptosis have all been shown to accompany cisplatin-induced acute renal failure in rats [94].

Thus, both free radical generation and depletion of antioxidants have been demonstrated in kidney in response to cisplatin administration. Similarly, it has been reported that attenuation of endogenous antioxidant production is a key mechanism by which cisplatin causes oxidative stress in the ear. Ototoxicity observed with cisplatin has a high incidence, may be acute or delayed, is irreversible, and no preventative treatments are available. Histopathologically, degenerative effects of cisplatin have been noted in outer hair cells in the organ of Corti, spiral ganglion cells, and marginal cells of the stria vascularis [95, 96]. It has been suggested that increased ROS generation relevant to ototoxicity in response to cisplatin may result from upregulation of nicotinamide adenine dinucleotide phosphate oxidases (NOX-1 and NOX-4). Exposure of immortalised HEI-OC1 auditory hair cells to cisplatin was associated with increased expression of NOX isoforms and cytotoxicity, whilst cisplatin administration to mice increased NOX expression in the cochlea. Conversely, inhibition of NOX using siRNA was associated with decreased ROS production and caspase 3 activation in HEI-OC1 cells, whilst exposing organ of Corti explants to (nonspecific) NOX inhibitors protected against cisplatin-induced hair cell loss [97]. These data highlight some of the molecular mechanisms that may underpin ROS generation in the ear. It has also been shown that cisplatin treatment in rats is associated with depletion of cochlear antioxidants glutathione peroxidase and glutathione reductase, elevations in SOD and catalase activities, and acute ototoxicity [98].

Given the putative role for redox imbalance in cisplatin-induced ototoxicity, it is unsurprising that a number of otoprotectors have been proposed with a view to developing a clinical strategy to mitigate the risk of hearing damage in patients. Again, several agents have been used in nonclinical studies including L-*N*-acetylcysteine, vitamin E, allopurinol and salicylate andamifostine(reviewed in [99]). However, translating protective effects from experimental systems to man may be difficult, as evidenced by data from randomised clinical trials in whichamifostinewas administered to patients receiving cisplatin: there was no compelling evidence thatamifostineprotected against ototoxicity [100, 101].

## 4. Opportunities for the Future: Personalized Health Care

The examples above indicate the potential for ROS generation, perturbations in oxidant homeostasis, and mitochondrial dysfunction to contribute to clinically relevant drug side effects. Recently, it has been suggested that it may be possible to use ROS measurements to predict the potential for chemical phototoxicity [102]. However, for the toxicities highlighted above, there is clearly interindividual difference in severity of toxicity and susceptibility. Is there future potential to identify the causes of individual differences and does this suggest opportunities for personalised health care? There are data to suggest pharmacogenomics may provide important insights.

Cisplatin-induced ototoxicity appears to depend not only on dose, as there are marked interindividual variations in toxicity in patients receiving similar cumulative doses of cisplatin [103]. Other factors are considered important, and it has been hypothesised that genetic variation may be a key component in determining susceptibility to the effects of cisplatin. For example, in a study involving 173 survivors of testicular cancer, genetic variants of glutathione S-transferase (GST) are described as a key determinant of cisplatin-induced ototoxicity: the GSTM1 polymorphism is described as detrimental and ^105^Val-*GSTP1* described as protective [104]. The work ongoing to identify a possible genetic component in cisplatin ototoxicity has been reviewed recently [105]. Given the efforts to identify specific single nucleotide polymorphisms and the use of genome-wide analysis to give insights into which population(s) may be most vulnerable to this side effect, perhaps it will be possible in the near future to identify susceptibility to cisplatin-induced ototoxicity based on genetics and to use this to manage risk of hearing loss in patients.

There is evidence of variants in proteins associated with the transport and metabolism of Dox, such as the SLC22A16 transporter [106] and SOD2 [107]. Consequently, genetically determined differences in cellular accumulation and Dox-induced redox imbalance may be relevant to clinical outcomes of Dox therapy, including toxicity. However, to date, a genotype that could be used to characterise a patient subset less susceptible to Dox-induced toxicity has not been identified [108]. If such a genotype could be identified, it may well include numerous genetic variations. Recently, a relationship between BRCA2 status and susceptibility to Dox-induced cardiac damage has been suggested [109]. BRCA2, a tumour-suppressor gene, encodes a protein involved in repair of chromosomal damage and is an indicator of increased risk of breast and ovarian cancer. In knock-out mice lacking BRCA2 specifically in cardiomyocytes, Dox exposure resulted in increased cardiotoxicity, as indicated by increased levels of cytochrome c release, p53 accumulation, and cardiomyocyte apoptosis [109]. In addition to directly determining apoptotic fate, p53 may both up- and downregulate ROS production: in cases of severe cellular stress, p53 may activate pro-oxidant genes leading to elevations in ROS [110]. Interestingly, the BRCA2 conditional knock-out mice themselves did not show an adverse cardiac phenotype, suggesting the cardiac effects observed were not due to BRCA2 dysfunction but suggesting BRCA2 status is a determinant of susceptibility to Dox-induced toxicity. Given the opportunity to screen prospectively for BRCA2 deletion, if these animal data translate to man, there is an opportunity to identify patient populations at increased risk of anthracycline-induced toxicity. Overall, these examples illustrate potential for genetic differences to determine susceptibility to the toxicity of two drugs for which oxidative stress may be a key contributor to adverse events.

## 5. Concluding Remarks

The examples presented here illustrate the potential for oxidative damage to contribute significantly to toxicities in man. However, though Dox, cisplatin, and AZT are well-characterised molecules and the clinical adverse effects are well established, the exact mechanisms by which ROS may induce their toxic effects are not fully established. The contribution of oxidative stress to the emerging safety profile of newer drugs remains largely unknown. It is clear that more data are required to provide insight into individual susceptibility to specific ROS-dependent mechanisms of toxicity. Understanding individual differences of this type and the potential for redox effects to manifest as toxicities is increasingly valuable not just for existing therapies but for tailoring clinical drug development.

## Figures and Tables

**Table 1:** Examples of toxicities associated with drug-induced oxidative stress.

Therapeutic class	Drug	Example toxicities	Evidence for oxidative stress	
Antineoplastic (anthracycline)	Doxorubicin	Cardiac toxicity	Reduction of doxorubicin to free radical increases ROS in cardiomyocytes. Lipid peroxidation, mitochondrial dysfunction, apoptosis	[111, 112]
Antiretroviral	AZT	Skeletal myopathy, cardiac toxicity	Increased ROS and NOS (peroxide and peroxynitrate). Overexpression of superoxidase dismutase/catalase protects against toxicity, apoptosis	[86, 113]
Anti-inflammatory	Diclofenac	Nephrotoxicity, hepatotoxicity	Oxidative stress generated by a cation radical or redox cycling of intermediates derived from hydroxylation. Multifactorial perturbations in mitochondrial dysfunction	[114, 115]
Analgesia	Paracetamol	Hepatotoxicity	Formation of reactive metabolite, depletion of glutathione, activation of proapoptotic proteins. Mitochondrial dysfunction, inflammation	[116]
Antineoplastic (platinum)	Cisplatin	Nephrotoxicity, ototoxicity	Increases in superoxide anion, hydrogen peroxide, and hydroxyl radical. Depletion of antioxidants GSH-peroxidase and GSH-reductase. Mitochondrial dysfunction, apoptosis	[93, 97, 99, 117]
Antipsychotic	Chlorpromazine	Dermal toxicity (due to phototoxicity)	Generation of singlet oxygen and superoxide in response to UVA/B irradiation	[102]
